# Supplementary material for: Transcription and splicing dynamics during early Drosophila development
Source: RNA. 2022 Feb;28(2):139–61. doi: 10.1261/rna.078933.121 (PMC8906543; doi:10.1261/rna.078933.121)
Supplement: Supplemental Material [file supp_28_2_139__DC1.html]

Transcription and splicing dynamics during early Drosophila development — Supplemental Material 

# Transcription and splicing dynamics during early *Drosophila* development

## Supplemental Material

- Supplemental\_Fig\_S1.pdf
- Supplemental\_Fig\_S2.pdf
- Supplemental\_Fig\_S3.pdf
- Supplemental\_Fig\_S4.pdf
- Supplemental\_Fig\_S5.pdf
- Supplemental\_Fig\_S6.pdf
- Supplemental\_Fig\_S7.pdf
- Supplemental\_Methods.pdf
- Supplemental\_Table\_1.xlsx
