# Supplementary material for: Transcription and splicing dynamics during early Drosophila development
Source: RNA. 2022 Feb;28(2):139–61. doi: 10.1261/rna.078933.121 (PMC8906543; doi:10.1261/rna.078933.121)
Supplement: Supplemental Material [file supp_078933.121_Supplemental_Fig_S1.pdf]

A

Dataset	Raw reads	Uniquely Mapped after Merge
Early_2-3h_S5P_rep1	62,891,575	12,959,070
Early_2-3h_S5P_rep2	56,917,697	3,610,607
Late_4-6h_S5P_rep1	65,865,258	21,564,673
Late_4-6h_S5P_rep2	75,909,131	27,375,998
Late_4-6h_S5P_rep3	71,923,876	11,987,034
Late_4-6h_S2P_rep1	77,838,463	38,595,780
Late_4-6h_S2P_rep2	64,862,996	23,137,529
Late_4-6h_S2P_rep3	76,719,886	17,912,504

B

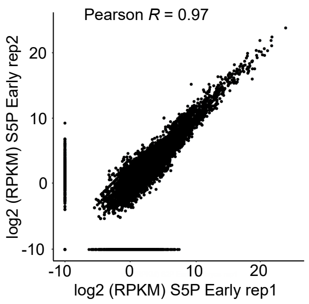

C

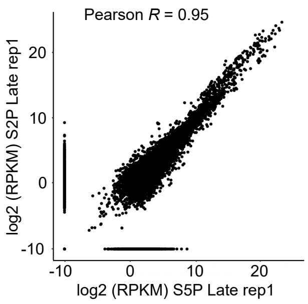

D

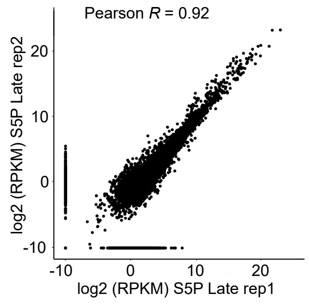

E

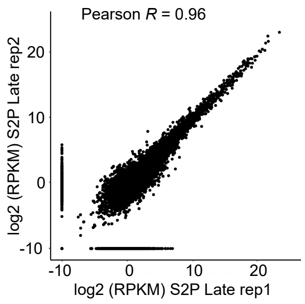

F

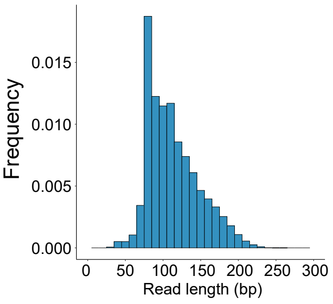

G

Dataset	Number of nascent reads	Nascent reads / Uniquely mapped reads (%)
Early_2-3h_S5P_rep1	7,984,411	61.6
Early_2-3h_S5P_rep2	2,430,876	67.3
Late_4-6h_S5P_rep1	13,413,045	62.2
Late_4-6h_S5P_rep2	13,777,880	50.3
Late_4-6h_S5P_rep3	8,505,056	71.0
Late_4-6h_S2P_rep1	23,153,659	60.0
Late_4-6h_S2P_rep2	12,926,707	55.9
Late_4-6h_S2P_rep3	10,356,743	57.8

H

Dataset	min	mean	max	25th percentile	50th percentile	75th percentile
Early_2-3h_S5P_rep1	35	99.6	293	74	81	119
Early_2-3h_S5P_rep2	35	94.3	293	72	81	110
Late_4-6h_S5P_rep1	35	114.2	293	86	108	135
Late_4-6h_S5P_rep2	35	102.0	294	80	87	119
Late_4-6h_S5P_rep3	35	88.4	294	76	81	89
Late_4-6h_S2P_rep1	35	107.0	294	81	96	128
Late_4-6h_S2P_rep2	35	111.1	294	81	102	132
Late_4-6h_S2P_rep3	35	109.0	293	83	105	130

Supplemental Fig. S1. (A) For each *d*NET-seq library prepared, the number of total reads and of uniquely aligned reads is indicated. (B) Density of uniquely aligned reads per gene (RPKM in log2 scale) for two *d*NET-seq/S5P biological replicates from early embryos (Pearson's correlation, R = 0.97). (C) Density of uniquely aligned reads per gene (RPKM in log2 scale) from late embryos analysed by *d*NET-seq/S5P and *d*NET-seq/S2P (Pearson's correlation, R = 0.95). (D, E) Density of uniquely aligned reads per gene (RPKM in log2 scale) for two *d*NET-seq/S5P and *d*NET-seq/S2P biological replicates from late embryos (Pearson's correlation, R = 0.92; R = 0.96). (F) Histogram of the read lengths in *d*NET-seq/S5P data from late embryos. (G) For each *d*NET-seq library generated, the number of uniquely aligned reads corresponding to nascente transcripts is indicated. (H) Length of sequenced nascent RNA (in nucleotides).
