# Supplementary material for: Transcription and splicing dynamics during early Drosophila development
Source: RNA. 2022 Feb;28(2):139–61. doi: 10.1261/rna.078933.121 (PMC8906543; doi:10.1261/rna.078933.121)
Supplement: Supplemental Material [file supp_078933.121_Supplemental_Fig_S2.pdf]

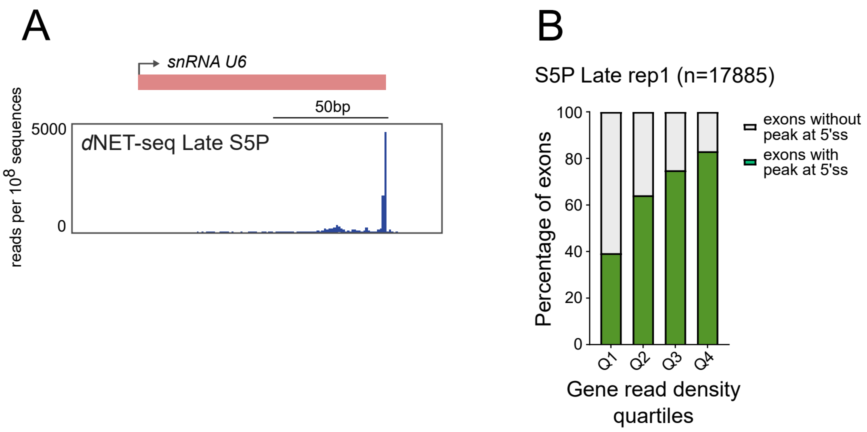

Supplemental Fig. S2. (**A**) *d*NET-seq/S5P profile over the U6 snRNA gene in the late dataset (replicate 1). (**B**) Frequency of peaks corresponding to splicing intermediates (green) detected by *d*NET-seq/S5Pon exons of genes expressed in late embryos. Genes were grouped into quartiles (Q) based on their *d*NET-seqread density.
