# Supplementary material for: Transcription and splicing dynamics during early Drosophila development
Source: RNA. 2022 Feb;28(2):139–61. doi: 10.1261/rna.078933.121 (PMC8906543; doi:10.1261/rna.078933.121)
Supplement: Supplemental Material [file supp_078933.121_Supplemental_Fig_S5.pdf]

A

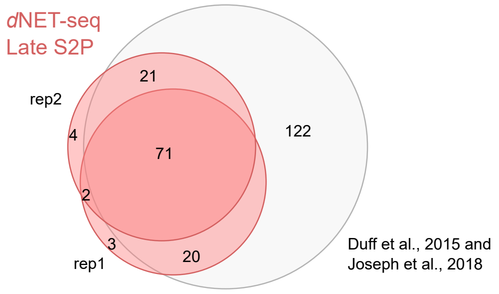

Supplemental Fig . S5. **(A)** Venn diagram comparing RPs identified in two *d*NET-seq/S2P biological replicates and in previously reported studies (Joseph et al. 2018; Duff et al. 2015).
