# Supplementary material for: Transcription and splicing dynamics during early Drosophila development
Source: RNA. 2022 Feb;28(2):139–61. doi: 10.1261/rna.078933.121 (PMC8906543; doi:10.1261/rna.078933.121)
Supplement: Supplemental Material [file supp_078933.121_Supplemental_Fig_S6.pdf]

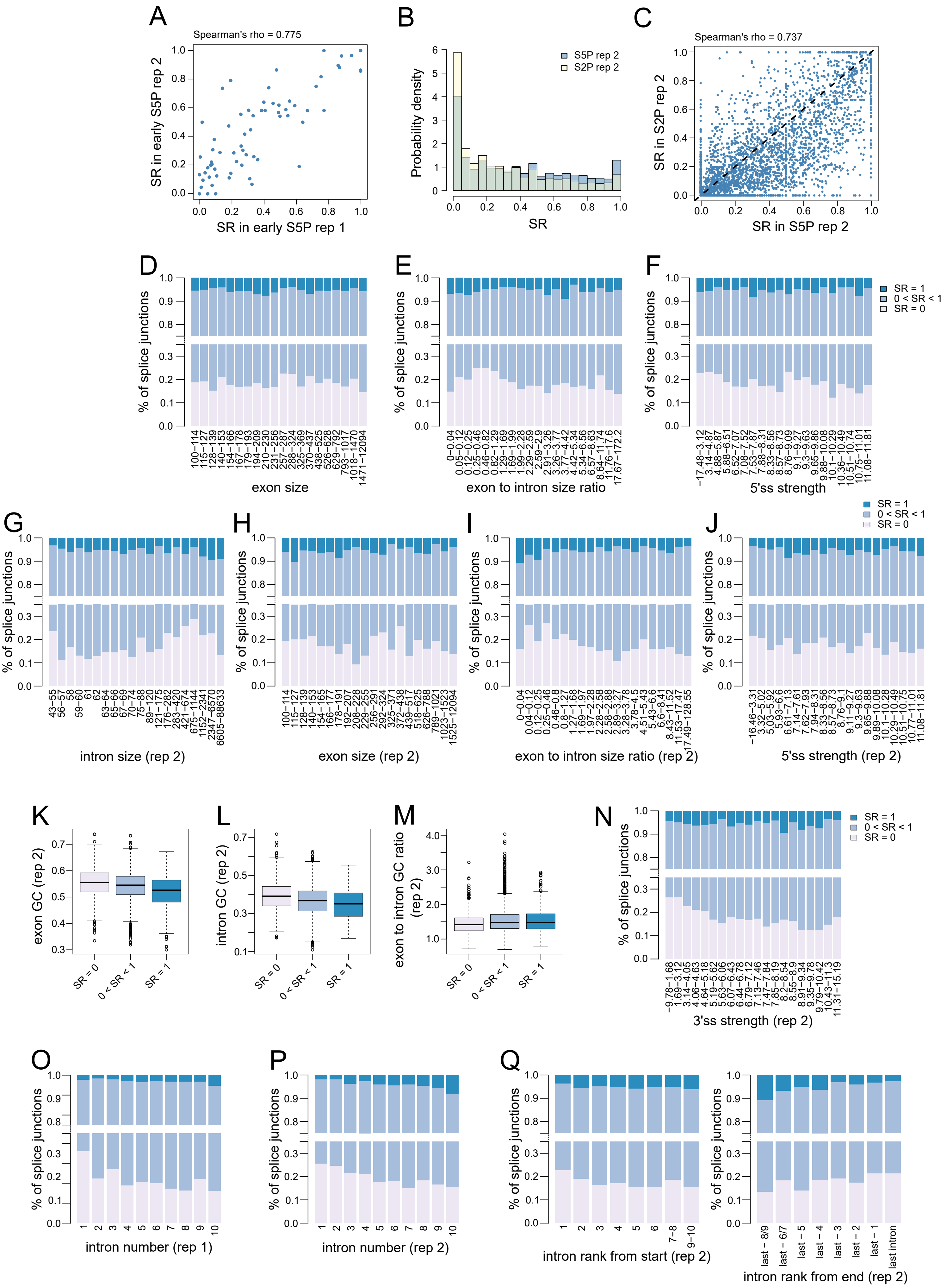

A
Spearman's rho = 0.775
SR in early S5P rep 2
SR in early S5P rep 1
B
S5P rep 2
S2P rep 2
Probability density
SR
C
Spearman's rho = 0.737
SR in S2P rep 2
SR in S5P rep 2
D
% of splice junctions
exon size
E
exon to intron size ratio
F
SR = 1
0 < SR < 1
SR = 0
5'ss strength
G
intron size (rep 2)
H
exon size (rep 2)
I
exon to intron size ratio (rep 2)
J
5'ss strength (rep 2)
K
exon GC (rep 2)
L
intron GC (rep 2)
M
exon to intron GC ratio (rep 2)
N
3'ss strength (rep 2)
O
intron number (rep 1)
P
intron number (rep 2)
Q
intron rank from start (rep 2)
intron rank from end (rep 2)
last − 8/9
last − 6/7
last − 5
last − 4
last − 3
last − 2
last − 1
last intron

Supplemental Fig. S6. In all panels, unless otherwise specified, only introns from transcriptionally active genes where the downstream exon is a fully coding internal exon at least 100 nt long were included. In addition, enough spliced/unspliced reads had to end within the first 100 exonic nucleotides that obtaining a splicing ratio (SR) of 0 or 1 by chance alone was highly unlikely (see the Methods section for details). For genes expressed in late embryos, this threshold was 10 reads for both replicates. For pre-MBT genes, it was 14 for replicate 1 and 9 for replicate 2. For S2P, a threshold of 10 was used for better comparability with S5P data. (A) SR values estimated in two biological replicates of *d*NET-seq/S5P datasets for pre-MBT genes in early embryos (Spearman correlation, ρ = ~0.775, P ~ 1.348 * 10-14). (B) Distribution of SR values for *d*NET-seq/S5P (N = 4510) and *d*NET-seq/S2P (N = 4373). (C) SR values estimated in replicate 2 of *d*NET-seq/S5P and *d*NET-seq/S2P from late embryos (Spearman correlation, ρ = ~0.737, P < 2.2 * 10-16; N = 3295). (D-Q) SR values for *d*NET seq/S5P data (replicate 1 and 2) plotted out against several gene architecture parameters. For sample sizes and statistical tests, see Supplementary Table 1. In (D-J, N-Q), the bin ranges have been set so that intron numbers would be as equal possible between bins.
